# Revised Global Typhoid Vaccination Policy

**DOI:** 10.1093/cid/ciy927

**Published:** 2019-02-15

**Authors:** Adwoa D Bentsi-Enchill, Joachim Hombach

**Affiliations:** Department of Immunization, Vaccines and Biologicals, World Health Organization, Geneva, Switzerland

**Keywords:** typhoid fever, typhoid conjugate vaccines, immunization policy, vaccination strategies, WHO vaccine position paper

## Abstract

Typhoid fever is a continuing public health problem in many low- and middle-income countries; however, routine vaccination as a recommended control strategy has not been implemented in the past in most countries. Greater understanding of the typhoid fever burden, the increasing threat of antimicrobial resistance, and licensure of a new generation of typhoid conjugate vaccines (TCVs) were instrumental in paving the way for the World Health Organization (WHO) to issue a revised global policy on typhoid vaccines in March 2018. The new policy follows evidence-based recommendations by the WHO Strategic Advisory Group of Experts on immunization for routine and catch-up vaccination with TCVs and highlights considerations for universal, risk-based, or phased vaccination strategies in different settings. Further, the first WHO-prequalified TCV and Gavi funding for eligible countries make vaccination a realistic control strategy for many affected countries, especially if combined with improved water and sanitation services, strengthened surveillance systems, and appropriate antimicrobial treatment.

An estimated 11 to 21 million cases and more than 128 000 deaths from typhoid fever occur annually across many low- and middle-income countries, primarily in South and Southeast Asia and sub-Saharan Africa [1–3]. Effective control strategies against typhoid, caused by *Salmonella enterica* serovar Typhi, include access to safe water, adequate sanitation, personal and food hygiene, health education, appropriate antimicrobial treatment, and vaccination. While water, sanitation, and hygiene (WASH) strategies are powerful tools for typhoid control, the major financial investments required for infrastructure development and maintenance limit WASH solutions in the short to medium term in most typhoid-endemic countries [4, 5]. Weighed against WASH, vaccination is more affordable to governments, does not require substantial behavioral change, and provides a shorter-term control strategy. Here, we describe the context and procedure for the recent revision of the global typhoid vaccination policy by the World Health Organization (WHO).

WHO is mandated to provide global vaccine policy recommendations based on a transparent and systematic evidence review process by the Strategic Advisory Group of Experts (SAGE) on Immunization, an independent advisory committee that advises the WHO on the development of policy related to vaccines and immunization [6, 7]. WHO then issues global policy through position papers that represent its official position on vaccines or vaccine-related issues that have an international public health impact [8].

In 2008, WHO updated its policy on the parenteral unconjugated Vi polysaccharide and oral live attenuated Ty21a typhoid vaccines and emphasized their programmatic use to control endemic and epidemic typhoid fever where the disease remained a significant public health problem. Public health use of the Vi polysaccharide vaccine in 2 large demonstration projects in Asia, in 2003–2004 and 2010–2012, provided evidence of vaccine effectiveness and safety, feasibility of community- and school-based delivery strategies, and acceptability of the vaccine in the target populations [9, 10]. However, in the 5 years (2009–2013) following the 2008 global policy recommendations, very limited vaccine uptake in routine immunization programs occurred in typhoid-endemic countries in the WHO South-East Asia region and Western Pacific region where a review was conducted [11]. Of note, that review found only 1 other example of large-scale typhoid vaccination (outside of the demonstration projects) initiated since the updated policy; in Fiji, a successful mass vaccination campaign with the Vi polysaccharide vaccine was conducted in 2010 in high-risk areas following a category 4 cyclone [12]. Further, there was no significant change in the trend of typhoid vaccination in endemic countries in the rest of the decade.

Several key developments over the last decade refocused attention on the potential for routine vaccine use as a critical public health tool, especially when integrated with WASH and other strategies, for the control of typhoid fever. Crucially, data from the Typhoid Surveillance in Africa Program showed moderate to high disease incidence in some sub-Saharan Africa countries, rivalling rates previously described in parts of Asia [13]. These gains in understanding of typhoid burden have contributed to defining the global and regional priorities for typhoid control.

Second, there has been a growing body of evidence about the threat of antimicrobial-resistant strains of *Salmonella* Typhi, including spread of the H58 haplotype, which has been responsible for much of the recent and current spread of resistant strains [14] and emergence of additional resistant haplotypes [15]. Concerns about a potential looming public health crisis with respect to “untreatable typhoid fever” and the possibility of reversal to preantibiotic case fatality rates were heightened in recent months following a report that described a *Salmonella* Typhi H58 strain with resistance to the traditional first-line antibiotics of ampicillin, chloramphenicol, and trimethoprim-sulfamethoxazole, as well as additional resistance to fluoroquinolones and ceftriaxone, in an ongoing outbreak in Pakistan [16].

Third, the long-anticipated availability of a newer generation of typhoid conjugate vaccines (TCVs) moved closer to reality with licensure in India of 2 Vi-tetanus toxoid conjugate vaccine products in 2008 and 2013. An earlier Vi-rEPA conjugate vaccine had provided extensive clinical trial data that demonstrated safety, immunogenicity, and clinical efficacy of 89.0% (95% confidence interval, 76.0, 96.9) over 46 months follow-up in Vietnamese children aged 2–5 years [17]. However, the Vi-rEPA vaccine was not licensed, and delays in TCV clinical development coupled with lack of funding support and apparent limited interest by decision makers in endemic countries to use the Vi polysaccharide and Ty21a vaccines meant that no real progress could be made toward a revised vaccination policy in the last decade. The first TCVs to receive national licensure therefore signalled a breakthrough toward a possible routine childhood typhoid vaccination strategy. Furthermore, TCVs have the additional value of longer duration of protection compared to existing vaccines.

Focus on typhoid conjugate vaccines necessitated a review of the (hitherto limited evidence on) age-specific burden in infants and children aged <2 years and the key considerations for the optimum schedule and delivery strategies for that age group. Revision of the typhoid vaccine policy followed the standard SAGE process for the development of vaccine recommendations, which incorporates review of the best available evidence on the clinical and epidemiologic characteristics of the targeted disease in different settings; vaccine characteristics and performance; and immunization, economic, health-system, legal, and ethical considerations, as well as social values and preferences regarding the target vaccine. This evidence review is combined with assessment of the quality of evidence on critical policy questions and, where needed, expert interpretation and judgment on specific programmatic questions to guide the resulting public health decision-making [7]. As with other vaccines, the typhoid vaccine policy development relied and built on specific reviews of data by other technical advisory bodies to WHO, such as the Global Advisory Committee on Vaccine Safety [18], and the Immunization and Vaccine-related Implementation Research Advisory Committee [19]. SAGE issued recommendations for TCVs (and updated those for the unconjugated Vi polysaccharide and Ty21a vaccines) in October 2017 [20], and the revised WHO policy was released in March 2018 [21].

The new WHO policy recommendations reemphasize programmatic use of typhoid vaccines for the control of typhoid fever and specify that among the available typhoid vaccines, TCV is preferred at all ages in view of its improved immunological properties, suitability for use in younger children, and expected longer duration of protection. Recommendations for routine and catch-up vaccination as well as the specific considerations for national decisions on the preferred vaccination strategy (universal, risk-based, or phased) are laid out in the WHO position paper [21].

With the first WHO prequalification of a TCV [22] and a decision by Gavi, the Vaccine Alliance, to provide funding support to eligible countries [23], a firm pathway to routine TCV use in the populations at most risk is set. Much work remains, particularly for national decision-makers and immunization providers to leverage the recent momentum to demonstrate and benefit from the real value of vaccination to reduce the burden of typhoid fever. Further, efforts must be made to combine vaccination with improved water and sanitation services, strengthened surveillance, and appropriate antimicrobial treatment. Equally crucial are the ongoing studies to further improve knowledge on the burden of severe typhoid [24–27] as well as research by the Typhoid Vaccine Acceleration Consortium to evaluate the effectiveness and impact of TCV in reducing typhoid burden in endemic countries [28].

## Notes

***Author Contributions.*** A. B. E. and J. H. wrote the manuscript. The development of WHO vaccine policy relies on SAGE on immunization and other advisory bodies as described here.

***Supplement Sponsorship.*** This supplement was sponsored by Coalition against Typhoid, Sabin Vaccine Institute, Bill & Melinda Gates Foundation.

***Potential conflicts of interest.*** A. B. E. and J. H. are staff members of the WHO, which receives grants from the BMGF to support typhoid-related and other work. All authors have submitted the ICMJE Form for Disclosure of Potential Conflicts of Interest. Conflicts that the editors consider relevant to the content of the manuscript have been disclosed.
